# Molecular Motors and Apical CFTR Traffic in Epithelia

**DOI:** 10.3390/ijms14059628

**Published:** 2013-05-03

**Authors:** Dmitri V. Kravtsov, Nadia A. Ameen

**Affiliations:** 1 Department of Pediatrics/Gastroenterology & Hepatology, School of Medicine, Yale University, New Haven, CT 06520, USA; E-Mail: dmitri.kravtsov@yale.edu; 2 Cellular and Molecular Physiology, School of Medicine, Yale University, New Haven, CT 06520, USA

**Keywords:** CFTR, trafficking, myosins, microtubules, actin transport

## Abstract

Intracellular protein traffic plays an important role in the regulation of Cystic Fibrosis Transmembrane Conductance Regulator (CFTR) chloride channels. Microtubule and actin-based motor proteins direct CFTR movement along trafficking pathways. As shown for other regulatory proteins such as adaptors, the involvement of protein motors in CFTR traffic is cell-type specific. Understanding motor specificity provides insight into the biology of the channel and opens opportunity for discovery of organ-specific drug targets for treating CFTR-mediated diseases.

## 1. Introduction

### 1.1. CFTR in Organs and Tissues

Cystic Fibrosis Transmembrane Conductance Regulator (CFTR) is a member of the ABC transporter-class of ion channels [1] that is expressed in multiple organs and tissues [2]. CFTR is the main epithelial chloride channel that mediates fluid secretion by creating a flux of chloride ions and accompanying water transport across the epithelium [3,4]. This anion-selective channel is required for the normal function of epithelia lining the airways, intestinal tract, pancreatic ducts and sweat glands [5]. The genetic disease Cystic Fibrosis (CF) results from mutations in CFTR that lead to deficient/dysfunctional CFTR in a variety if epithelial tissues. CF is associated with exocrine pancreatic insufficiency, an increase in sweat NaCl concentration and male infertility; however the major cause of morbidity and mortality is pulmonary disease [6]. Loss of CFTR from airway epithelial cells also leads to altered regulation of other ion channels, changes in inflammatory pathways and the composition of airway surface liquid [7,8]. CFTR channels are the most important mediators of anion secretion from the brush border membrane (BBM) of crypt and villus enterocytes in the intestine [3,4]. Absence of functional CFTR on the enterocyte BBM leads to intestinal obstruction while increased CFTR on the enterocyte BBM leads to excessive fluid secretion and diarrhea. Thick acidic mucus accumulation on epithelial surfaces is a pathogenetic feature of CF disease. Although CFTR is not present in mucus producing goblet cells, its presence in neighboring epithelial cells is important for mucus hydration and alkalinization that is facilitated by CFTR-mediated HCO3^−^ transport [9]. Bicarbonate loosens and expands mucus [10] to facilitate its movement along the epithelial surface. Thus, CFTR serves important functions in different organs and tissues in regulating electrolyte and fluid balance, mucus viscosity and luminal pH.

### 1.2. Regulation of CFTR by Trafficking

A number of key steps and pathways including biosynthesis, glycosylation, exocytic transport to the apical plasma membrane (PM), endocytosis, recycling and degradation are critical in the “life-cycle” of CFTR [11]. Exocytic insertion of CFTR into the apical PM, endocytosis and recycling comprise the apical trafficking pathway. CFTR has a long life cycle with a half-life of greater than 24 h [12]. This scenario allows for the existence of an apical recycling pool of CFTR in the vicinity of the plasma membrane that can maintain surface levels of CFTR. In epithelial tissues such as the intestine, the subapical pool of CFTR is important for stress responses (*E. coli*, *Vibrio* Cholera infection) by rapidly increasing fluid secretion through recruitment of mature CFTR into the plasma membrane [13]. Thus, CFTR can be regulated through variations in the rate of biosynthesis (hours to days), apical recycling or recruitment of mature CFTR molecules from subapical pools into the plasma membrane. Recruitment into the apical plasma membrane occurs within minutes [14,15], providing rapid control of CFTR channel activity that can be finely regulated by local, tissue and cell-type specific molecular switches [16]. CFTR exerts its function in epithelial cells when it is localized in the plasma membrane (PM). Apical plasma membrane trafficking of CFTR has been shown to be dependent on the SNARE vesicle fusion protein syntaxin 3 [17]. Once on the PM, CFTR is resident for some variable period, following which it undergoes constitutive or stimulated clathrin-dependent endocytosis into EEA 1 or Rab 5 positive vesicles [10,18,19]. CFTR can then enter a Rab 11 pool of recycling endosomes [19–21] undergo re-insertion into the PM or be targeted to late endosomes and lysosomes for degradation via a Rab7 pathway [19]. Endocytosis and recycling allows for accumulation of CFTR in close proximity to the plasma membrane without excessively burdening the secretory machinery. In addition, endocytosis efficiently regulates CFTR channel activity by modulating the number of CFTR channels on the PM. Protein kinase A (PKA) [22] and Protein kinase G (PKG) [23] activate the channel by phosphorylation of its R domain and stimulate exocytosis and PM recruitment of CFTR [17,24,25]. A number of excellent reviews have been written on CFTR traffic and regulation [11,16,26], however, one aspect of CFTR traffic remains overlooked. How CFTR moves between cellular trafficking compartments has not been addressed. In the present review, we summarize data from studies that investigated the roles of microtubule and actin motor proteins in CFTR localization and function in tissues and cells. This review is focused on motors involved in the apical recycling of CFTR.

## 2. Microtubule-Dependent Transport in CFTR Trafficking

Physical translocation of CFTR molecules between cellular domains and compartments requires the involvement of molecular motors. Microtubules are well-characterized tracks for intracellular delivery [27] and the role of microtubule transport in the apical localization and function of CFTR was initially studied using microtubule inhibitors. Doses and timing of treatment with inhibitors, as well as resulting changes in CFTR currents were included in this review because these data are useful for understanding the effects of motor protein inhibitors on specific cell types, developmental stages and site of action on CFTR trafficking.

### 2.1. Intestinal Epithelium

In 1992 Grotmol *et al*. reported on the involvement of microtubule-dependent transport in CFTR trafficking [28]. The authors examined the role of microtubules (MT) in Cl^−^ secretion from rat distal colon following stimulation with 10 μM prostaglandin E2 and 10 mM theophylline. The MT inhibitors colchicine, nocodazole and taxol all inhibited the stimulated ΔIsc and reduced the 60-min integrated secretory response to PGE2 and theophylline (integral of ΔIsc) by 39%–52% (Table 1). In colchicine-treated tissues, PGE-stimulated Cl^−^ absorption was significantly reduced by 25% compared to untreated controls, however, no effect on sodium transport was observed. This study demonstrated the importance of an intact microtubule transport for CFTR-mediated Cl^−^ currents in distal rat colon.

In 1994 Morris *et al.* [29] used 5 μg/mL Brefeldin A (BFA) a transport protein inhibitor (ER to Golgi) to treat polarized monolayers of HT-29 colonic carcinoma-derived cells and reported that anion efflux was not affected by BFA. However, pre-incubation with BFA for 12 h decreased the ΔIsc responses elicited by 10 μM forskolin (FSK) by 50%–60% (Table 1) and the observed half-life of inhibition ranged from 8 h in 6-day old cultures to 13 h in 48-days old cultures.

Two years later, in 1996 Tousson and colleagues used polarized colonic T-84 cells to examine the dependence of apical CFTR recruitment on microtubules and microfilaments following stimulation with cAMP or Ca^2+^ agonists [30]. Using immunofluorescence approaches, they found that treatment with 33 μM nocodazole (3 h) reduced FSK (10 μM) induced apical recruitment of CFTR to about 40% of vehicle-treated cells while pretreatment of the cell monolayer with cytochalasin D (10 μM, 1 h) had no effect on the FSK-evoked apical recruitment of CFTR (as measured by relative fluorescence intensity). The rate constant of ^125^I efflux from cytochalasin D-treated T84 cells in response to stimulation with 10 μM FSK increased 3-fold in control and 3.7-fold in 10 μM cytochalasin D treated cells. In contrast, 30 s. stimulation with 10 μM FSK caused a 2-fold increase in relative fluorescence intensity, the effect that was prevented with nocodazole treatment. In addition, nocodazole increased sub-apical relative fluorescence intensity as compared to FSK-treated cells.

In 2003 our group examined how cAMP-dependent exocytosis and vesicle traffic regulate CFTR and fluid transport in rat jejunum *in vivo* [25]. In that study, ligated intestinal loops were treated with 0.1 mM primaquine + 1 mM 8-bromo-cAMP (8-BrcAMP), 10 μg/mL nocodazole + 1 mM 8-Br-cAMP, 1 mM 8-Br-cAMP for 2 h. The findings were as follows: in the control loops, 2 h treatment with 1 mM 8-Br-cAMP induced translocation of subapical CFTR to the apical PM. Co-administration of primaquine (0.1 mM) reduced apical CFTR immunofluorescence and increased subapical CFTR signal, and this effect was associated with a 35% reduction in the fluid response of the primaquine-pretreated loops. These findings are consistent with the reported effects of primaquine in preventing vesicle fusion with the plasma membrane [31]. Pretreatment with 10 μg/mL nocodazole resulted in a reduction in subapical to apical shift of CFTR immunofluorescence in response to cAMP, although to a less degree compared to primaquine; fluid secretion in response to cAMP treatment was reduced by 33%. Our observations confirmed the role of vesicle traffic in CFTR localization and function in response to cAMP and demonstrated that although microtubule transport was involved, other factors including vesicle fusion were important to CFTR trafficking in the intestine. The difference in response suggests involvement of other cellular networks such as actin filaments.

### 2.2. Airway Epithelium

Loffing and colleagues [32] used the airway epithelial cell line Calu-3 to examine CFTR ion transport and a possible role of microtubules following stimulation with 100 μM CPT c-AMP. These authors reported that neither pre-treatment with nocodazole (33 μM) nor taxol (10 μM) for 3 h prior to cAMP stimulation had an effect on CFTR current (Table 1). Immunofluorescence localization studies demonstrated that neither drug altered the cellular localization of CFTR. The authors proposed that Isc response to the cAMP reflected the long half-life (>3 h) of CFTR channels on the PM and suggested that the disruption of MT has an effect on vesicle delivery to the apical pole, but no effect on the recycling of early endosomes [33].

### 2.3. Renal Epithelium

Morris *et al*. reported in 1998 that disruption of microtubules inhibited arginine-vasotocin (AVT) -stimulated Cl^−^ secretion, but not Na^+^ reabsorption in A6 *Xenopus* kidney cells that have been used extensively to study amiloride-sensitive Na^+^ transport [34]. They inhibited microtubule or actin-dependent transport in A6 cells using 100 μM colchicine or 33 μM nocodazole (30 min at 4 °C followed by 3 h at 26 °C) or 500 nM cytochalasin E (1 h at 26 °C). The authors found that microtubule disruption inhibited a 0.1 μM AVT-stimulated secretory Cl^−^ current but did not prevent activation of amiloride-sensitive Na^+^ transport. Basal Isc was approximately three times higher in control cells compared to nocodazole-treated cells (Table 1). Despite the overall reduction in Isc response to AVT, the relative response in nocodazole-treated cells was greater than that of control (3.9 *vs*. 2.5 fold, respectively). In colchicine-treated cells, the relative increase in response to stimulation was roughly the same for treated vs. control (1.9 *vs*. 2.0, Table 1). One very interesting finding from this study was the biphasic nature of AVT-stimulated Isc curve in A6 cells. The initial Isc response that was sensitive to nocodazole treatment disappeared in the low Cl^−^ Ringer preparations and was abolished by treatment with 300 μM glibenclamide. Immunofluorescence labeling revealed that nocodazole treatment reduced apical CFTR levels and induced basolateral and intracellular accumulation of the antigen. Treatment with AVT had no effect on CFTR localization in nocodazole-treated cells, while apical translocation of the antigen was observed in control cells. The authors concluded that AVT-induced translocation of CFTR was not completely interrupted by nocodazole treatment. In contrast, nocodazole treatment had no effect on the sodium channel that was examined in parallel with CFTR in this study. The authors concluded that microtubule-dependent transport is important for proper cellular localization of CFTR as well as electrophysiological components of CFTR-mediated response to the treatment with AVT. However, the relative roles/contribution of microtubule vs. actin transport was not addressed. The authors suggested that membrane trafficking responses to AVT could vary based on the developmental stage of the epithelium.

Moyer *et al.* used a stable transfected MDCK kidney cell line to examine trafficking of GFP-tagged CFTR [35]. The authors examined the mechanism of stimulated Cl^−^ secretion through CFTR and concluded that in this cellular model, CFTR trafficking, unlike phosphorylation, does not play a significant role [35]. Their conclusions were based on the following results: 5–7 h pre-treatment with 33 μM colchicine had no effect on stimulated Isc following cAMP cocktail (100 μM CPT-cAMP, 100 μM sobutylmethylxanthine, and 20 μM forskolin, see Table 1). No effect on basal or steady state Isc was observed. Quantitative immunofluorescence experiments and apical surface biotinylation revealed a small decrease (90% of control level) in biotinylated apical membrane CFTR. These authors attributed the observed effect of microtubule disruption on CFTR recycling, which contradicts other reports in the literature [33]. Long PM half-life of CFTR was suggested as an explanation for the observed persistence of apical biotinylated surface CFTR and Cl^−^ current in the presence of MT disruption.

### 2.4. Summary: Microtubule-Dependent Delivery

The specific microtubule motors involved in the movement of CFTR has not been addressed directly. In 2002 Johnston, Illing and Kopito examined the role of cytoplasmic dynein in the assembly of aggresomes [36]. In this study, GFP-ΔF508CFTR, used as a marker for aggresome formation, localized to the same cellular areas as both kinesin and dynein in stably transfected HEK293 cells. While ΔF508CFTR is a mis-folded mutant that does not undergo apical recycling [37], these data still argue in favor of the involvement of microtubule motors at least in the very early (immediately post-Golgi) and late (degradation) stages of CFTR trafficking. The association of ΔF508CFTR with dynein was also reported by Burnett and Pittman [38]. MT involvement in CFTR exocytic transport has been clearly demonstrated in the intestinal epithelium, but this was not as evident in airway and kidney cells. Moreover, inhibition of MT transport does not lead to the complete inhibition of CFTR-mediated Cl^−^ currents. Total inhibition of vesicle movement appears to be more effective then inhibition of MTs. Involvement of the MTs may depend on the developmental stage of the cell. The specific motors associated with microtubule-dependent transport of wild type CFTR were never examined in sufficient details.

## 3. Actin-Based Transport in CFTR Trafficking

Patch-clamp and short circuit current studies have suggested that actin may regulate Cl^−^ secretion in colonic, bronchial and CFTR-expressing heterologous cells [39–41]. Myosins are a family of actin-based motors that consists of more than 20 classes [42], of which members of myosin classes I, II, V, VI, and VII were found in association with actin of brush borders [43,44], and myosins I, V, VI, VII were observed in ciliated epithelia [45]. Thus, for the purpose of this review, data on the role of actin-based motors in CFTR trafficking is subdivided in accordance with specific cell type and class of myosin.

### 3.1. Intestinal Epithelium

#### 3.1.1. Myosin Vb and Exocytosis of CFTR

The first study providing evidence of involvement of MyoVb in the trafficking of CFTR was published 2000, when Ameen and Salas [46] examined tissues from patients with the genetic disease Microvillus Inclusion Disease (MID). MID is a devastating childhood disease that presents with intractable diarrhea [47] leading to water losses in excess of 140 mL/kg/day [48]. Mutations in the MyoVb gene [49,50], lead to absence of a functional protein in enterocytes that is associated with MID. MID is characterized by unique histologic features in the intestine; enterocytes contain subapical inclusions possessing microvilli (MI’s) that are accompanied by severe disorganization of the brush border [46]. We reported that in human MID affected intestine, CFTR was significantly reduced at the apical PM, mis-localized with a diffuse distribution in the apical cytoplasm below the terminal web and accumulated in MI’s. In contrast to apical proteins, basolateral membrane proteins such as Na^+^-K^+^-ATPase, were not mis-localized in MID-affected enterocytes, suggesting a specific defect in the apical trafficking pathway that may target a late step in exocytosis.

#### 3.1.2. Myosin VI Regulates Endocytosis of CFTR in the Intestine

In 2007 Ameen and Apodaca examined the apical membrane distribution, endocytosis and anion transport function of CFTR in the intestine of Myosin VI (Myo6) deficient Snell’s Waltzer (sv^−/−^) mice [51]. Those studies demonstrated that absence of Myo6 led to alterations and disorganization of the intestinal brush border. In heterozygote control mouse intestine, CFTR co-localized with Myo6 in the intestinal crypts and apical domain of villus enterocytes; however in the Myo6 sv^−/−^ intestine, apical CFTR was five-fold higher compared to the controls. In Myo6 sv^−/−^ small intestine, apical endocytosis was delayed over 0 to 1 min and was markedly reduced with only 10% of surface CFTR endocytosed between 1 and 5 min. This contrasted with endocytosis in the control intestine where more than 45% of surface CFTR was internalized. Overall, the rate of apical endocytosis was reduced by 70% in Myo6 sv^−/−^ intestine. Luminal treatment of Myo6 sv^−/−^ closed jejunum loops to the cGMP agonist heat-stable enterotoxin (STa) (0.5 μM for 30 min) resulted in a statistically significant four-fold higher fluid accumulation compared to controls, consistent with an increase in surface expression of functional CFTR in Myo6 sv^−/−^ jejunum.

#### 3.1.3. Myosin Ia is a Brush Border-Specific Regulator of CFTR

Myosin Ia (Myo1a) is one of the most abundant proteins in the intestinal brush border [43] where it functions synergistically with Myo6 in the maintenance of enterocyte brush border structure [52]. In 2012 our group reported on Myo1a dependent traffic of CFTR into the brush border membrane using intestinal segments from Myo1a KO mice and the polarized intestinal Caco-2BBe cell model [18]. We observed a gradient of Myo1a expression, with maximum antigen levels detectable in the brush border decorating tips of the small intestinal villi, while Myo1a was not apically localized in the crypts. The absence of Myo1a resulted in failure of CFTR to traffic into the brush border of villus enterocytes and its accumulation in subapical vesicles. We demonstrated that this defect resulted from impaired CFTR exocytosis that involved accumulation of CFTR in a late exocytic syntaxin3-positive compartment. Ussing chamber experiments demonstrated that in the duodenum there was a marked decrease in anion secretion at baseline, such that the Isc in Myo1a KO tissue demonstrated only 100 ± 34.7 μamp/cm^2^ compared to 318 ± 46.5 μamps/cm^2^ in WT tissue. Furthermore, stimulation with 10 μM FSK increased Isc by 31.6 ± 13.7 μamps in the Myo1a KO tissue compared to 76 ± 13.6 μamps in tissue from WT animals. Currents in the colon were similar in the WT and KD. These data are consistent with involvement of Myo1a in CFTR traffic and function in the villus epithelium and the absence of Myo1a in crypts and colon. Co-immunoprecipitation studies performed using Caco-2_BB_e cell lysates demonstrated that CFTR and Myo1a form a complex. Using purified isolated brush borders from Myo1aKO animals and surface biotinylation in the Caco-2BBe cells lacking Myo1a expression, these studies demonstrated that the Myo1a-dependent defect in CFTR localization is specific to the brush border, the site of CFTR function in the enterocytes. Myo1a appears to exert specificity for brush border membrane proteins as it regulates CFTR and Sucrase-Isomaltase localization [53] but not Alkaline Phosphatase and PAT1 (SLC26A6).

### 3.2. Airway Epithelium

#### 3.2.1. Myosin VI Facilitates Endocytosis of CFTR

By the early 2000s several classes of myosin motors, including a minus-end endocytic motor myosin VI (Myo6) were characterized [45,54,55] and in 2004 Swiatecka-Urban and colleagues [56] examined a role for Myo6 in CFTR endocytosis. The authors employed HEK293 cells overexpressing CFTR and polarized human airway epithelial cells (Calu-3). Co-immunoprecipitation demonstrated that CFTR forms a complex with Myo6 in Calu-3 lysates; the endocytic proteins clathrin and Dab2 were identified in the same complex. Overexpression of a dominant-negative Myo6 tail in HEK293 cells resulted in more than 50% increase in the membranous population of CFTR. The authors demonstrated that increase in number of CFTR molecules residing on the PM is the result of selectively impaired endocytosis. Treatment of HEK293 cells with 2 μM cytochalasin D did not affect fluid phase endocytosis, but the fraction of CFTR endocytosed in 5 min. was reduced from ~9% of the total membrane CFTR to ~1.25%, a significant difference of 86%.

#### 3.2.2. Myosin Vb Regulates Exocytosis of CFTR in the Airway Epithelial Cells

In 2007 Swiatecka-Urban and colleagues [20] examined the importance of Myosin Vb (Myo5b) for CFTR trafficking in the airway epithelium. The authors found that in polarized airway epithelial Calu-3 cells Myo5b formed a complex with CFTR while Myo5c isoform did not. Following silencing of Myo5b expression, apical CFTR levels dropped by 50% in airway epithelial CFBE410 cells transfected with WT-CFTR and by 40% in the Δ508-CFTR transfected cells (cultured at 27 °C), while the level of intracellular CFTR remained unchanged. Knock down (KD) efficiency was ~55% in WT-CFTR cells and ~70% in Δ508-CFTR cells. Reduction in surface CFTR levels was confirmed in the experiments using a dominant-negative Myo5b tail. Ussing chamber experiments demonstrated that silencing of Myo5b inhibits the 20 μM forskolin-stimulated short-circuit current in a CFTR_inh172_-sensitive manner. Myo5b affected recycling of CFTR without perturbing endocytosis. Studies performed by this group suggest that Myo5b acts in conjunction with the Rab GTPase, Rab11a.

### 3.3. Summary on Myosins

Unlike microtubule-associated motors, much attention was paid to the role of myosins in regulating CFTR during the last decade. Myo5, Myo6 and Myo1a were found to have precise roles in the apical trafficking of CFTR both in the airway and/or intestinal epithelium. Research on Myo6 has identified its role as the most significant motor underlying clathrin-based endocytosis of CFTR, a fundamental step in the lifecycle of the channel in respiratory and intestinal epithelia. Data on involvement of the Myo5b motor in the exocytosis of CFTR in the intestinal and airway epithelium are particularly interesting from the perspective of relative importance of microtubule and actin transport in the apical delivery of CFTR. Precisely where Myo5b exerts its action in the exocytic process is unknown, and whether the site of Myo5b action is the same in different cell types is unknown. For example, it seems plausible that in the airway epithelium Myo5b becomes important early in exocytosis, thereby reducing dependence on microtubule transport compared to the intestine.

Data on Myo1a suggest that there could be two types of specificity for myosin function, site-directed specificity, as Myo1a regulates CFTR in the microvilli, and channel-specificity, since Myo1a selectively affects CFTR while other apical transporters are unaffected. In addition, Myo1a is specifically localized to mature enterocyte brush borders [57], where it regulates CFTR exocytosis. CFTR is more abundant in crypt cells where it regulates anion secretion from the brush border membrane. While Myo6 is present and regulates CFTR endocytosis in both crypt and villus enterocytes, the specific myosin responsible for apical exocytosis in the crypt has not been identified.

## 4. Conclusions

A growing body of literature continues to support cell and tissue specificity of CFTR trafficking [58]. Several studies demonstrated tissue-specificity of CFTR binding partners [18,20,21,58,59]. As an example, robust studies confirmed that Rab11 involvement in subapical trafficking of CFTR requires tissue specific isoforms (Rab11a in the airway epithelium *vs*. Rab11b in the intestinal epithelium). Similarly, tissue specific regulation of CFTR was demonstrated for syntaxin SNARE proteins [60]. This review summarized available data on the involvement of motor proteins in CFTR trafficking. Although the number of published studies on this subject is limited, important conclusions can be drawn.

In the intestine, cyclic nucleotide-stimulated trafficking of CFTR requires both microtubule and actin transport, while microtubules appear to play a minor role in agonist-stimulated trafficking of CFTR in airway cells. Significant controversy remains in drawing conclusions from studies of CFTR trafficking in non-polarized and cell models lacking endogenous CFTR and the wide spectrum of agonists used in the studies of the stimulated trafficking of CFTR that may produce cell type specific effects (Table 2).

While agonists may share common pathways, the effects of these drugs on the individual proteins participating in the trafficking of CFTR may vary.

**Figure 1:**
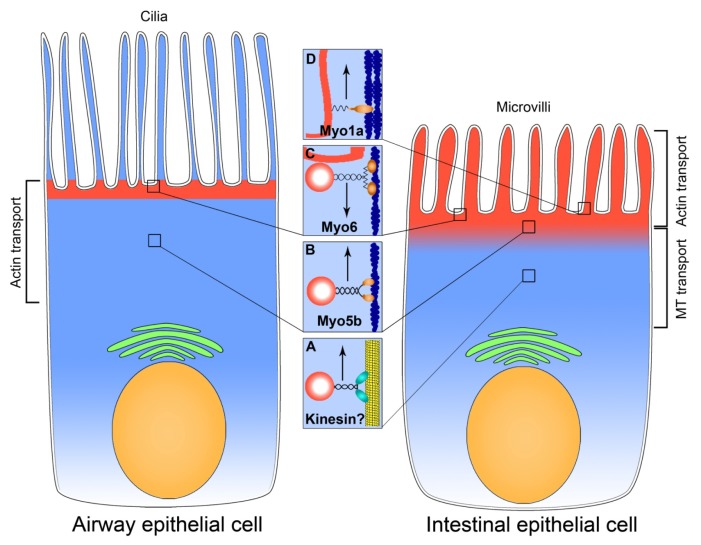
Motor transport systems involved in CFTR exocytosis in the airway and intestinal epithelium. CFTR (red) is localized to the apical domain of airway and intestinal epithelial cells. In airway epithelium, CFTR decorates the apical plasma membrane in the inter-ciliary space and subapical region, while in the intestinal epithelium CFTR is in the subapical cytoplasm, intermicrovillar and microvillar membranes. Brackets show the predominant type of transport associated with the apical delivery of CFTR. Nuclei are shown in beige, cytosol in light blue and Golgi apparatus in green. The insets **A**–**D** indicate the motors that move CFTR in the particular region of the cells, highlighted with squares. (**A**) MT-associated motor kinesin moves CFTR-associated vesicle up the microtubule in the intestinal epithelial cell; (**B**) Myo5b moves exocytic CFTR-associated vesicle on microfilaments in airway and intestinal epithelial cells; (**C**) Myo6 facilitates endocytosis of CFTR in airway and intestine; (**D**) Myo1a is important for the exocytosis and, probably, tethering of microvillar membrane-inserted CFTR in the enterocyte. Microtubule is shown in yellow, microfilaments in dark blue and PM-inserted CFTR in red. Upward arrows facing indicate exocytosis, downward arrow–endocytosis.

Figure 1 represents a diagram summarizing the data on microtubule *vs*. actin delivery of CFTR to the apical plasma membrane of intestinal and airway epithelial cells. The difference in the type of transport may be linked to the distribution pattern of apical CFTR. In enterocytes, CFTR is found along the length of microvilli and in the intermicrovillar domain [15], while in airway epithelial cells CFTR is localized to the interciliary region but is not on the cilia membrane [61]. While regulated exocytosis of CFTR plays a prominent role in fluid secretion in the intestine, its importance in the airway epithelium remains controversial [62,63]. Review of available data on CFTR translocation in the airway epithelium suggests that respiratory epithelial cells rely more on actin motors for exocytic transport of CFTR. This scenario would account for the lack of observed effects of microtubule inhibitors on Cl^−^ transport through CFTR in airway cells. The complex and highly developed actin cytoskeleton in the apical pole of enterocytes and the need for bidirectional translocation of CFTR in this region of the cell necessitates a more complex regulation that can account for preferential actin-based transport in this region, while microtubules transport CFTR in the intracellular space.

Cell type specificity of motors in CFTR regulation opens up the possibility for organ-selective regulation of CFTR in diseases. For example, since Myo1a is an intestine-specific myosin, modulation of Myo1a may be useful in designing treatments for diarrheal diseases. Nevertheless, major gaps remain in our understanding of CFTR regulation by molecular motors. The role of kinesins, as well as other members of the myosin family such as myosin II and myosin VII, has not been investigated. How CFTR switches from microtubules to microfilaments during exocytosis is also not understood. Very little or nothing is known regarding the role of motors in non-intestinal and non-airway CFTR expressing cells, such as kidney and pancreatic ducts [2].

Neither the actin or microtubule transport system can be considered more important than the other. Future studies must focus on further dissection of the role that tissue and cell-type specific isoforms of motor proteins play in CFTR trafficking. Extrapolation of data from non-epithelial and over-expressing cell models must be interpreted with caution, until the mechanisms of the delivery are confirmed to be identical to those in the target organs and cells.

## Figures and Tables

**Table 1:** Summary of published results of MT and pharmacologic inhibitors’ effects on Cl^−^ currents, in various epithelial cells.

		N 30–33 μM	Col 100 μM	Col 33 μM	T, 20 μM	T, 10 μM	BFA, 5 μg/mL	Control
Grotmol, 1992 Rat colon 10 μM PGE_2_ + 10 μM Theophylline	Basal	35 (24–46)	20 (2–37)		36 (21–40)			30 (21–48)
	Stimulated	34 (24–45)	37 (29–49)		33 (20–60)			56 (46–93)

Morris, 1994 HT-29 intestinal cells 10 μM FSK	Stimulated						6.3 ± 1.0	20 ± 1.9

Morris, 1998 A6 kidney cells 0.1 μM AVT	Basal	5.1 ± 0.4	3.2 ± 0.8					15.3 ± 2.7
	Stimulated	19.7 ± 1.9	6.3 ± 1.7					38.2 ± 4.1

Moyer, 1998 MDCK kidney cells cAMP mix	Stimulated			11.9 ± 0.4				10.2 ± 0.7

Loffing, 1998 Calu3 airway cells	Stimulated	61.9 ± 3.1				44.8 ± 2.0		60.5 ± 1.8

N, nocodazole; Col, colchicine; T, taxol; BFA, brefeldin A; numbers are expressed as ΔIsc, μA/cm^2^.

**Table 2:** The summary of pharmacological agents used for CFTR stimulation in the study of microtubular motors.

Group	Cell type	Agent used
Grotmol, 1992	Rat colon	10 μM PGE_2_ + 10 mM theophylline
Morris, 1994	HT-29	10 μM FSK
Tousson, 1996	T-84	10 μM FSK
Loffing, 1998	Calu3	100 μM CPT-cAMP
Morris, 1998	A6	0.1 μM AVT
Moyer, 1998	MDCK	100 μM CPT-cAMP + 100 μM sobutylmethylxanthine + 20 μM FSK
Ameen, 2003	Rat jejunum	1 mM 8-BrcAMP
